# Biased image cropping and non-independent samples

**DOI:** 10.1186/s12915-016-0307-9

**Published:** 2016-10-04

**Authors:** John F. Y. Brookfield

**Affiliations:** School of Life Sciences, University of Nottingham, University Park, Nottingham, NG7 2RD UK

## Abstract

Any figure in a research article will typically represent only a small portion of the total data gained by a researcher for that experiment, and it is therefore key that the figure accurately reflects what was found overall. Furthermore, if individual observations form clusters with differing mean properties, those individual observations would not represent independent samples from the populations being compared. In this example, the question of how to fairly represent and treat image data is addressed.

There are bodies that can be observed in mouse cells through the labelling with green fluorescent protein of one of their protein components. But the function of these bodies is not known and their number per cell is highly variable. The mouse cells are found in clusters, with each cluster being surrounded by a membrane. An experiment is carried out in which a gene thought to be involved in repressing the creation of these bodies is inactivated. In presenting the data initially, the authors submit Fig. 1, cropped images of their confocal data, showing the numbers of the bodies (green circles or ovals) in each of nine cells of a single cluster in either the control (repressor protein wild type; Fig. 1a) or treatment (repressor protein inactivated; Fig. 1b). The authors present cropped images as these “show most clearly the effect of the gene inactivation”. While the reviewers for the paper can see that there are more spots in the treatment than in the control cells, they ask for a statistical test to confirm this and the authors reveal that the nine cells in the treatment cluster have an average of five spots per cell, whereas the nine cells of the control cluster have an average of one spot per cell. This is highly significant (t = 4.80, 16df, *p* < 0.001). But one reviewer also asks to see a larger group of cells and the authors submit now the uncropped images, showing four clusters per treatment (Fig. 2). The reviewer feels that the initial group of cells was not telling the whole story as the numbers of spots in these treatment cells were considerably higher than the number of spots in the rest of the cells. But the authors counter by showing that, even if all the 36 treatment cells are compared to the 36 control cells, the mean number of spots in the treatment cells is still significantly higher (three versus two; t = 2.372, 70df, *p* = 0.020).

**Fig. 1:**
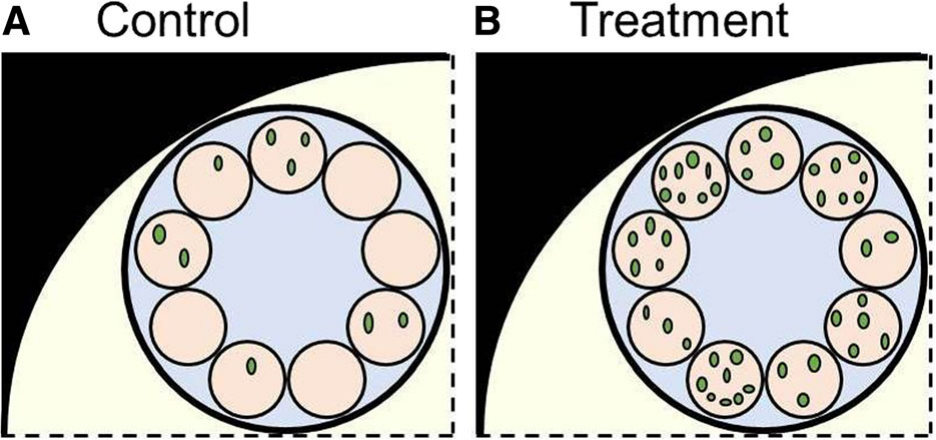
Cropped images of the authors’ confocal data, showing the distribution of green fluorescent protein spots in nine control (**a**) or treatment (**b**) cells in a single cluster

**Fig. 2:**
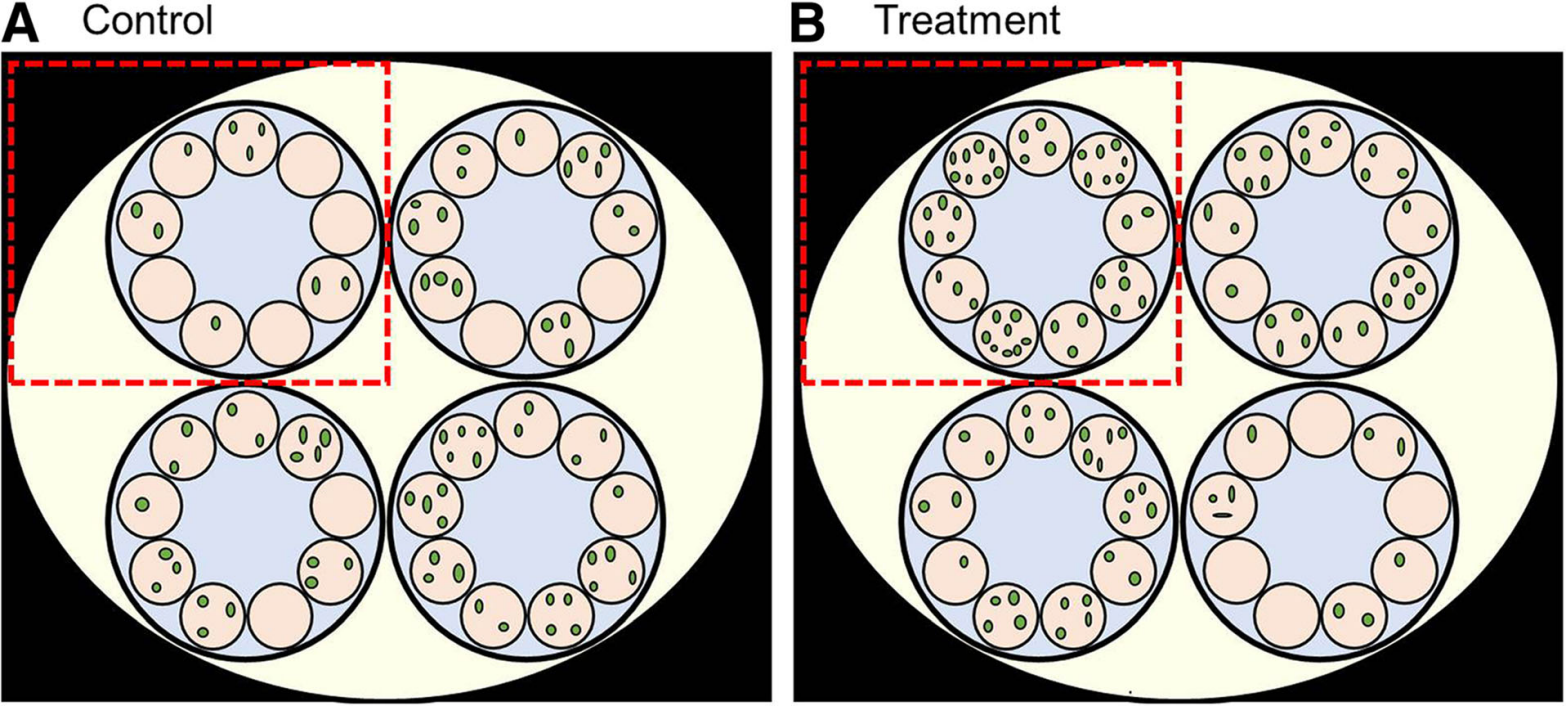
The uncropped version of Fig. 1 showing the distribution of green fluorescent protein spots in nine control (**a**) or treatment (**b**) cells in four clusters. The *red rectangles* in each panel show the cropped image included in the first submission of the paper

However, the apparent variation in spot numbers between the clusters of cells means that the individual cells are non-independent samples from the overall populations of treatment and of control cells. If the 36 cells in each panel of Fig. 2 are analysed as four clusters of nine, these four clusters of cells show significant variation (ANOVA F = 9.846, 3, 32 df, *p* < 0.001) in the treatment and in the control (ANOVA F = 3.718, 3, 32 df, *p* = 0.021) cells. This invalidates the test of 36 treatment cells versus 36 control cells. If the means of the four treatment clusters observed are compared to the means of the four control clusters observed, there is no significant difference (t = 1.095, 6df, *p* = 0.315). Cells from other parts of the organism and from multiple individual mice should be compared statistically.

